# Predictive Memory and the Surprising Gap

**DOI:** 10.3389/fpsyg.2012.00420

**Published:** 2012-10-17

**Authors:** Felipe De Brigard

**Affiliations:** ^1^Department of Psychology, Harvard UniversityCambridge, MA, USA

Clark (in press) has offered a forceful defense of the “hierarchical prediction machine” (HPM) approach to the brain. Roughly, HPM suggests that brains are in the business of making sense of incoming information by generating top-down models aimed at providing the optimal fit for the input data. A better fit between the model and the data minimizes prediction error, which Clark – following Friston (e.g., Friston, 2010) – construes as tantamount to reducing surprisal, i.e., “the sub-personally computed implausibility of some sensory state given the model of the world” (p. 17). Notwithstanding the breadth of his defense, Clark’s case is entirely built upon research on perception, attention, and action, all of which are on-line cognitive processes. With practically no mention of offline cognition, the theoretical pretensions of the HPM approach, which Clark so vigorously defends as a “single unifying explanatory framework” (p. 61) in cognitive science, are questionable.

I suggest that this conspicuous absence might be partially remedied, at least for the case of remembering, by looking at recent Bayesian accounts of memory retrieval developed after Anderson’s Adaptive Control of Thought-Rational (ACT-R) model (Anderson and Milson, 1989; Anderson, 1990; Anderson and Schooler, 1991, 2000). Specifically, I suggest that the ACT-R model can be read as describing how memory retrieval attempts to minimize prediction error when finding the optimal memory given the costs of its retrieval and the organism’s current needs. Originally, the ACT-R model stated that remembering is a cognitive operation whose costs are offset by the gains attained when retrieval is successful. As such, our adaptive memory system would search for a particular memory as long as the probability of recovering it given our current needs is greater than the costs of its retrieval. The ACT-R model captures this insight in Bayesian terms thus: let *H_i_* be the hypothesis that a particular memory is needed during a particular context, and let *E* be the evidence for an element of said context. Then,

(1) $$P\left(H_{i}|E\right)\alpha P\left(E|H_{i}\right)P\left(H_{i}\right)$$

where *P*(*E*|*H_i_*) determines the likelihood ratio that *E* is the case given *H_i_* (i.e., the *context factor*), and *P*(*H_i_*) gives the prior probability that a particular memory will be needed (i.e., the *history factor*). For present purposes, two consequences that follow from this formulation are relevant. First, as Anderson and Milson (1989) remarked, given the multiplicity of elements present in a retrieval context, the likelihood ratio representing the context factor is best understood as the multiplicative product of all the likelihood ratios for every element of the context given *H_i_*^1^. As a result, certain contextual elements are going to be better cues than others (i.e., representing a larger positive contribution to the overall product), as it is the case with elements that were present in the context of encoding (Craik and Tulving, 1975).

The second thing to notice is that the prior probability, according to the ACT-R model, depends on the history of previous retrievals. Originally, Anderson and Milson (1989) noted that determining the history factor could be daunting, if not impossible, as one “would have to follow people about their daily lives, keeping a complete record of when they use various facts [and] such an objective study of human information is close to impossible” (p. 705). To get around this problem, Anderson and Schooler (1991) suggested extracting prior probabilities from the statistical distribution of existent databases that, according to them, would capture “coherent slices of the environment.” One such environmental database, for instance, contained 2 years worth of word usage in the *New York Times* headlines. They found that the odds that a particular word was used in a certain headline was inversely correlated to its having occurred in a previous headline, with the probability diminishing the more time had passed since its last usage. Importantly, Anderson and Schooler (1991) showed that this model could fit extant data on recency and frequency effects on memory retrieval remarkably well^2^. Taken together, the context and the history factors suggest that the probability that a certain memory will be needed in a particular context can be predicted from the probability that it has been needed in the recent past in relevantly similar contexts. From the point of view of Clark’s HPM approach then, context and history factors combine in a hierarchical model that tries to find the most predictable memory – i.e., that which minimizes prediction error – for a needed memory given a cue^3^.

Notwithstanding Anderson and Schooler’s impressive results, priors based on statistical distributions of limited environments do not seem to capture the full complexity of human memory retrieval. Recently, however, Hemmer and Steyvers (2009b, see also Hemmer and Steyvers, 2009a) tried a different tack. They obtained the prior probability of remembering the size of a certain object from the statistical distribution of participant’s responses on a norming phase, in which relative size judgments on a number of objects had to be performed. Thus, instead of determining the prior by collecting data from the participant’s size-judgment behavior before the study, they did it via generating a probability distribution from the participants’ judgments themselves. This model allowed them to predict with remarkable accuracy hits and false alarms in a recognition test, as participant’s responses approximated the means of the prior distribution for each item.

So far I’ve tried to draw parallels between ACT-R inspired Bayesian models on memory retrieval and Clark’s HPM approach as a way to show that his explanatory framework can be extended to an offline cognitive process such as remembering. But in so doing I intentionally drew a stark contrast between the way in which Anderson and Schooler obtained prior probabilities and likelihood rates, and the way in which Hemmer and Steyvers did, because I think this difference illustrates a difficult challenge for the HPM framework. According to Clark, although the HPM approach is primarily thought of as describing the way in which the brain aims at reducing surprisal at anon-agential level, these very same processes may help to understand the agent-level experience of surprise reduction – the experience of sensing a stimulus as the least surprising (“surprisal-ing”!, p. 47). But this agent/non-agent gap may be difficult to bridge. Indeed, Clark himself acknowledges this difficulty when he says:

“[T]here seems to be a large disconnect between ‘surprisal’ (the implausibility of some sensory state given a model of the world) and agent-level surprise. This is evident from the simple fact that the percept that, overall, best minimizes surprisal (hence minimizes prediction errors) ‘for’ the brain may well be, for me the agent, some highly surprising and unexpected state of affairs” (p. 46).

Nonetheless, Clark (in press) believes that the two levels “are easily reconciled” when one recognizes that what appears to the agent as a surprising event may just be, in reality, only improbable. The agent might not have been expecting to experience some mental content or another, but from the point of view of the brain, such a content may actually be perfectly predictable.

I find Clark’s response unsatisfying, for this surprise-surprisal gap – this “surprising gap” – between the agent and the non-agent levels is likely to occur more often than Clark assumes, and the frequency of this occurrence puts pressure on Clark to come up with a clearer explanation as to how HPM can in fact illuminate cognition at the agent-level. Consider the two approaches to generating prior probabilities and likelihood ratios mentioned above. In the case of Anderson and Schooler, the approach is agent-independent, as it involves collecting probability distributions of frequency responses that are independent of the subject’s own frequency-judgments. Likewise, priors generated from data at the neural level, such as those referenced by Clark in his essay, are also agent-independent. Conversely, Hemmer and Steyver’s approach is paradigmatically agent-dependent, as it involves generating a probability distribution from the participant’s own frequency-judgments. However, we have plenty of evidence showing that what we think is most frequent does not always correspond to what it is actually most frequent (Tversky and Kahneman, 1973, but see Manis et al., 1993). Moreover, the agent/non-agent mismatch that gives rise to this “surprising gap” may actually occur even when there is no experience of surprise at the agent-level. It may occur, for instance, when there is a prediction mismatch due to independent processes of prior updating at the agential and non-agential levels. As a result, although models with agent-independent priors may be equally good at fitting data as models with agent-dependent priors, they need not be, and it is an open empirical question whether or not they do – a question that cannot be simply dismissed on *a priori* grounds, as Clark does. So it seems to me that studying this surprising gap is itself an exciting avenue for future research. Why are there percepts that may appear surprising to the agent? What are the conditions under which surprise reduction meets surprisal reduction? Are false alarms in perception or in recognition memory better predicted with agent-dependent or agent-independent priors? These, I think, are all interesting questions worthy of being examined, and for which the HPM needs to find an answer if it really attempts to be a “unifying explanatory framework” for both agent and non-agent level cognitive phenomena^4^.

## Footnotes

^1^To reflect the fact that each element q_j_ of the context E has a baseline probability of being associated to any other element, such as i, the likelihood ratio would have to be modified thus:

(2) $$P\left(E|H_{i}\right)=\prod_{q_{j}\in E}P\left(q_{j}|H_{i}\right)$$

^2^Notably, Anderson (1993) proved that the ACT-R model for memory could be captured with the standard activation formulas of connectionist models. This fueled memory research on Bayesian approaches to neural networks, which draws yet another stark parallel with Clark’s HPM approach.

^3^Incidentally, ACT-R approach also dovetails nicely with one of the leading cognitive accounts of memory retrieval: fuzzy trace theory. According to this view, remembering consists in the reconstruction of a previously encoded experience via the mutual coordination of two processes: the retrieval of a general gist of the encoded event plus the reinstatement of its distinctive (i.e., verbatim) details (Brainerd and Reyna, 2001). Dual-route models that assimilate the general gist to the prior probability and the verbatim information to the context factor show how the fuzzy trace theory of memory retrieval can be computationally implemented (Steyvers et al., 2006; Steyvers and Griffiths, 2008). Also, future research may illuminate connections between these approaches and recent proposals on the adaptive role of constructive memory for predictions (Bar, 2009; Schacter and Addis, 2009; Schacter, in press).

^4^Many thanks to Timothy F. Brady, Shamindra Fernando, Justin Jungé, and Daniel L. Schacter for helpful comments.
